# Lineage Decision-Making within Normal Haematopoietic and Leukemic Stem Cells

**DOI:** 10.3390/ijms21062247

**Published:** 2020-03-24

**Authors:** Geoffrey Brown, Lucía Sánchez, Isidro Sánchez-García

**Affiliations:** 1 School of Biomedical Sciences, Institute of Clinical Sciences, University of Birmingham, Birmingham B15 2TT, UK; 2 School of Law, University of Salamanca, 37007 Salamanca, Spain; id00698454@usal.es; 3 Experimental Therapeutics and Translational Oncology Program, Instituto de Biología Moleculary Celular del Cáncer, CSIC/Universidad de Salamanca, 37007 Salamanca, Spain; isg@usal.es; 4 Institute of Biomedical Research of Salamanca (IBSAL), 37007 Salamanca, Spain

**Keywords:** leukaemia, stem cells, haematopoiesis, lineage decision making, oncogenes, cancer

## Abstract

To produce the wide range of blood and immune cell types, haematopoietic stem cells can “choose” directly from the entire spectrum of blood cell fate-options. Affiliation to a single cell lineage can occur at the level of the haematopoietic stem cell and these cells are therefore a mixture of some pluripotent cells and many cells with lineage signatures. Even so, haematopoietic stem cells and their progeny that have chosen a particular fate can still “change their mind” and adopt a different developmental pathway. Many of the leukaemias arise in haematopoietic stem cells with the bulk of the often partially differentiated leukaemia cells belonging to just one cell type. We argue that the reason for this is that an oncogenic insult to the genome “hard wires” leukaemia stem cells, either through development or at some stage, to one cell lineage. Unlike normal haematopoietic stem cells, oncogene-transformed leukaemia stem cells and their progeny are unable to adopt an alternative pathway.

## 1. Introduction

The mechanisms by which haematopoietic stem cells (HSCs) “choose” to develop towards a particular specialised cell are still unclear. The process of decision-making comprises of a number of steps, including the generation of a number of possible options, the selection of one option from the range and whether there is irrevocable closure of other options or do they remain latent/clandestine. There is also the matter of how a cell “remembers” and passes on a fate to its progeny. To move towards a model of these aspects to stem cell behaviour requires a better understanding of various factors including stochastic and ordered gene expression, the overall architecture of the network of the control components and the influence of external factors [1].

The lineage-affiliation and differentiation status of cells equates to both the repertoire of transcription factors that is available and chromatin structure governance of the accessibility of cis-regulatory elements. Individual transcription factors that drive the gene expression for cells to develop along a pathway can additionally close down other developmental options. These processes can therefore occur simultaneously (reviewed in [2]). For example, PU.1 activates myeloid-specific genes and represses erythroid-, innate lymphoid cell (ILC)-, and T-cell-affiliated genes in a concentration dependent manner. PU.1 therefore enforces myeloid and B-cell fates and directs progenitors away from erythroid, ILC and T-cell fates [3]. High levels of the erythroid Kruppel-like factor EKLF expression promote erythropoiesis whilst suppressing megakaryopoiesis, in part by repressing the level of Fli-1 message [4]. Much of the work in the field of epigenetics, that is non-DNA-encoded, modifications to the genome has focussed on how patterns of histone modification, DNA methylation and chromatin conformation affect the transition between accessible and inaccessible gene states and this, in turn, is important to cell differentiation. Heritable epigenetic marks are probably essential to the stepwise progression of differentiation and as a cellular memory to fix a particular pattern of gene expression. The social environment a cell resides in, particularly the exposure of HSCs to haematopoietic cytokines (see below), may establish epigenetic programs that are long-term [5] and therefore the interplay between the epigenome and external influences is probably crucial to cell decision-making and the progression of cell differentiation. To add to the diverse nature of controls, a change to the three-dimensional organisation of the nucleus as a whole influences the capacity for cell differentiation towards a specialised cell type [6].

## 2. The Nature of Haematopoietic Stem Cells

Investigators used various means to show that just one HSC is sufficient to engraft the entire haematopoietic system long-term into a recipient irradiated mouse. They included flow cytometry to purify HSCs, retroviral tagging of HSCs and suitable immune deficient recipient mice [7]. Notta and colleagues cell sorted CD49f+ve cells from cord blood HSCs and optimised the engraftment of single cells into a NOD-scid-IL2Rgc−/− (NSG) mouse by using intra-femoral injection. In one experiment, they observed multi-lineage chimerism in 5 of 18 mice (28%) and a lower frequency of transfer in the second experiment (14%) [8]. The observed frequency of multi-lineage chimerism is rather low and relates to either the inefficiency of xenotransplantation [9] or the heterogeneous nature of the HSCs purified (see below). Nevertheless, the above study showed that all of the lineage fates are open to HSCs at one time during their lifespan.

The cardinal properties of HSCs derived from transplantation studies are that they are pluripotent and self-renew. Since the 1980s, we have viewed HSCs as giving rise to the different blood and immune cells by means of HSCs that have selected to differentiate progressing stepwise via a series of binary decisions, and intermediate progenitor cells, towards an end cell type. This viewpoint has unpinned many of the tree-like lineage maps for haematopoiesis, for example, the “classic” lymphoid/myeloid dichotomy map [10].

Contrary to the longstanding properties of HSCs, investigators have argued that self-renewal, as revealed by transplantation experiments, is not solely a feature of HSCs nor a feature that occurs naturally in these cells [11]. Moreover, there is initiation of affiliation to a single cell lineage as early as within HSC. Indeed, adult human HSCs are a mixture of some pluripotent cells and many cells with lineage-affiliated gene expression signatures [12]. Similarly, mouse HSCs selectively express the receptors for cytokines that are lineage-affiliated regarding their role in ensuring the survival and cell proliferation of cells that belong to a cell lineage. The receptors include those for erythropoietin (Epo), macrophage colony-stimulating factor (M-CSF), granulocyte colony-stimulating factor (G-CSF), granulocyte/macrophage colony-stimulating factor (GM-CSF) and the fms-like tyrosine kinase 3 ligand (Flt3L) [13]. This individual expression of receptors is highly germane to decision-making because the cytokines can instruct the lineage choice of mouse haematopoietic stem and progenitor cells (HSPCs). M-CSF instructs myeloid fate in HSCs and macrophage fate in granulocyte/macrophage progenitors. G-CSF and GM-CSF provoke the generation of neutrophils from granulocyte/macrophage progenitors. Flt3L drives myeloid-lymphoid development of primitive mouse bone marrow cells, and suppresses the development of megakaryocyte and erythroid progenitors (reviewed in [14]).

**Figure 1:**
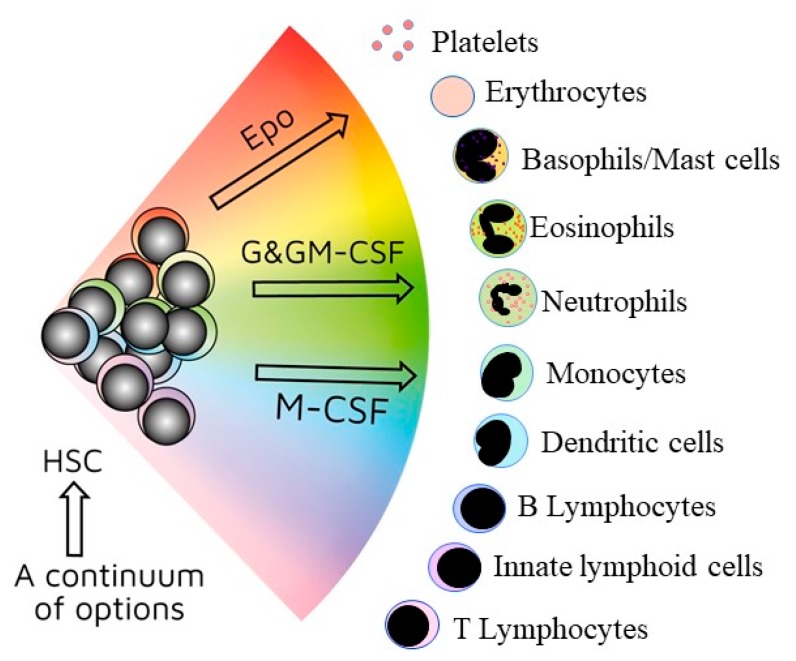
A continuum model of haematopoiesis. Haematopoietic stem cells (HSCs) “choose” a lineage from all options and are a mixture of cells with different lineage signatures (shown by the different colours). They and their progeny retain enough versatility to “step sideways” into a different pathway. Erythropoietin (Epo), granulocyte colony-stimulating factor (G-CSF)/granulocyte/macrophage colony-stimulating factor (GM-CSF), and macrophage colony stimulating factor (M-CSF), respectively, instruct erythroid, neutrophil, and monocyte fates within HSCs/haematopoietic progenitor cells.

In keeping with many new findings about the architecture of haematopoiesis, our view is that HSCs can directly choose one option from the entire spectrum of haematopoietic cell fates (Figure 1). Analysis of the transcriptomes of single haematopoietic progenitor cells (HPCs) as they differentiate placed the priming of fate potentials on a continuous transcriptional landscape with fate choice occurring earlier than previously thought [15]. We have placed particular cell lineages adjacent to one another in our continuum model, and evidence to support close relationships is described elsewhere [2]. For HSCs and HPCs that have selected a cell lineage, the other options remain latent/clandestine. In other words, they can still change their mind and “choose” an alternative developmental pathway (see below).

How might HSCs veer towards one versus another pathway? Halley and colleagues have argued that stochastic gene expression within the stem cell gene-regulatory network self-organises to a critical state leading to a cascade of expression to prime a fate [1]. Similarly, Hu and colleagues argued from early studies that examined single multipotent HSPCs, by RT-PCR, for the expression of lineage-affiliated genes that there is stochastic and low level “priming” of different lineage programs. They observed a “promiscuous” co-expression of cytokine receptor by single cells [16]. It is therefore interesting to speculate that cytokine receptor expression may be highly dynamic, based on fluctuating gene expression, and play a stochastic role in the precipitation of lineage affiliation. Intriguingly, the receptors for the instructive cytokines are positively auto-regulated by cytokine engagement. In this scenario, enforcement of a cell lineage affiliation is likely to occur once an HSC expresses the receptor and if the cytokine is available in the environment [14]. HSCs express the receptor for stem cell factor, to ensure their survival, and the “promiscuous” co-expression of a lineage-affiliated cytokine receptor may sound-out developmental options, to comply with the demand for a cell type, or provide a survival advantage if there is competition for stem cell factor for survival/cell expansion.

## 3. Crossing Barriers and “Stepping Sideways” to an Alternative Pathway

The intracellular barriers that separate highly distinct cell lineages/fate options are surmountable as to the four Yamanaka transcription factors Oct4/Sox2/Klf4/c-Myc reprogram somatic cells such as adult fibroblasts to induced pluripotent stem (iPS) cells [17]. OCT4 alone is sufficient to reprogram mouse neural stem cells to iPS cells [18]. Intrinsic reversibility is perhaps a little contrary to models for haematopoiesis that dictated that HSCs invariably develop along inflexible and the shortest routes to each end cell fate. However, and as above, alternative fates remain clandestine within HSPCs that have “chosen” a lineage and single-cell RNA sequencing of more than 1600 HSPCs provided insight to the availability of options as HSPCs differentiate. Nestorowa and colleagues used single-cell expression data to construct differentiation trajectories, leading to the conclusions that they are broad and, at any moment in time, cells do have the option of making a sideways movement rather than taking the shortest route to an end cell type [19]. In support of broad trajectories is that platelet production shares a pathway with the erythroid lineage, and at early stages with the pathways for mast cells, eosinophils and basophils [20].

Velten and colleagues constructed developmental trajectories by integrating single-cell RNA sequencing data with data from single cell cultures that revealed lineage potentials. The data support a continuum-based model of early haematopoiesis whereby HSCs gradually acquire lineage biases and commitment is a continuous process with barriers that separate lineages deepening gradually. Velten and colleagues brought to attention that small differences in developing cells in the expression of fate mediators might lead to the initiation of the barriers, favouring an early emergence of lineage-affiliation and within HSCs [21]. Of further interest is the developmental stage at which barriers are insurmountable to the extent that the instructive cytokines are not able to drive developing cells towards a fate that is different from the one first chosen (see below).

Ten percent of murine long-term repopulating HSC (LT-HSCs) express the megakaryocyte-associated surface antigen CD41 [22] and murine HSCs that express c-Kit at a high level are primed to differentiate towards platelets [23]. Around half of murine LT-HSC express the megakaryocyte lineage marker von Willebrand Factor (vWF) [24,25] as revealed using a transgenic (vWF)-GFP mouse model [26]. Whilst megakaryocyte-affiliated HSCs gave rise to platelet-biased reconstitution of irradiated mice, they were not restricted to repopulating platelets. Investigators observed a considerable “side-step” contribution to myeloid reconstitution and a low contribution to lymphoid reconstitution. As might be expected, biased HSCs are important to “an emergency” whereupon cells of a certain lineage are required because there was rapid recruitment of platelet-biased (vWF+ve) HSCs to replenish circulating platelets following acute depletion. Megakaryocyte-biased HSCs also produced platelets rapidly in the case of acute thrombocytopenia [27]. All of the above supports the view that lineage-biased/affiliated cells can give rise to cells of their “chosen” lineage in one circumstance and these and other cell types in a different circumstance, for example, an irradiated (empty-vessel) mouse. In other words, haematopoiesis is both dynamic and non-linear.

The barriers to developing haematopoietic cells moving from one lineage choice to another are somewhat “fluid” and overcome by lack of just one transcription factor together with treating cells with an appropriate cytokine as shown by studies of the B-cell lineage. B-cell lineage cells exclusively express the *Pax5* gene, encoding the transcription factor B-cell-specific activator protein (BSAP). *Pax5*-deficient (*Pax5^−/−^*) pro-B cell lines are not restricted in their lineage fate because they develop in vitro towards macrophages, dendritic cells, osteoclasts, granulocytes or natural killer cells when cultured with an appropriate cytokine. Culture of *Pax5^−/−^* pro-B cells on ST2 cells, which produce M-CSF, or with M-CSF (without stromal support and after culturing for 10–14 days on ST2 cells) led to macrophage differentiation. Terminal differentiation towards dendritic cells required GM-CSF instead of M-CSF. The cytokine TRANCE (also known as RANKL) controls the differentiation of osteoclasts and culture of the *Pax5^−/−^* pro-B cells on ST2 cells that ectopically expressed TRANCE gave rise to dendritic cells. Granulocyte differentiation required the presence of IL-6 and G-CSF and a small percentage of the cells differentiated, and ILC development required IL-2 and culture with stromal cells. Following reconstitution in mice, *Pax5^−/−^* pro-B cells gave rise to T cells. Restoration of Pax5 activity repressed this lineage versatility and therefore Pax5 plays a role to suppress alternative lineage choices in addition to facilitating B-cell development [28,29].

**Figure 2:**
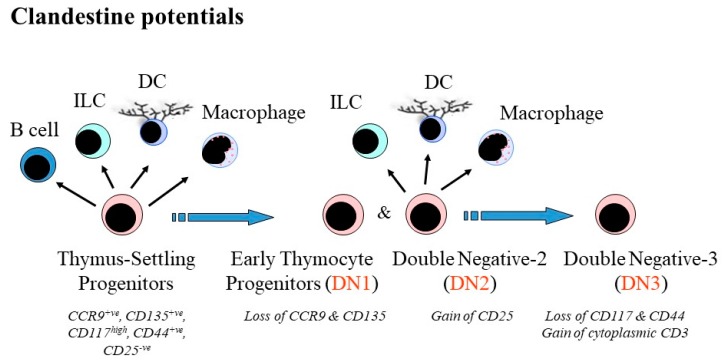
The availability of alternative pathways to developing thymocytes. Thymocytes that are well on their way to becoming T cells in the thymus can still give rise to macrophages, dendritic cells (DC), B cells and innate lymphoid cells (ILC). Macrophage colony-stimulating factor is required for the generation of macrophages. Culture of DN1 and DN2 cells in the presence of IL-7 and IL-2 led to the generation of ILC. IL-4 and IL-13 guide early thymocyte progenitors to develop towards DCs.

More mature progenitors are versatile. Double negative (DN2) thymocytes in the thymus are well on their way to becoming T cells but they can still give rise to macrophages, dendritic cells, B-cells and ILCs (Figure 2) [30,31,32]. Appropriate culture conditions are crucial to forcing DN1 and DN2 cells to step sideways. Culture of these cells on ST-2 stromal cells led to the generation of functional macrophages [31] and ST-2 cells produce a low level of macrophage colony-stimulating factor (M-CSF), which can instruct macrophage fate. Macrophage colonies did not arise from DN1 and DN2 cells when they were cultured on the M-CSF-non-secreting OP9 stromal cells. Culture of DN1 and DN2 cells in the presence of IL-7 and IL-2 led to the generation of ILCs, though IL-7 was not required to any large degree. IL-4 and IL-13 guide early thymocyte progenitors to develop towards dendritic cells with a CD8α+ve phenotype [32].

During their lifespan, some mature immune cells change the characteristics that affiliate them to a sub-type of cells. The different types of the mature CD4+ve effector include T helper 1 cells, T helper 2 cells (Th2), interleukin (IL) 17-producing T helper cells (Th17), follicular T helper cells (Tfh) and regulatory T cells (iTreg). Their sub-type functionality relates to each producing a different array of cytokines, for example, Th2 cells produce IL-4, IL-5, IL-13, IL-10 and IL-25 whereas iTreg produce IL-10, IL-35 and TGFβ. CD4+ve cells can switch from one to another type of CD4+ve cell and environmental signals drive the adoption of a new phenotype. Th2 cells can give rise to Tfh cells [33] and iTreg can convert to pro-inflammatory Th17 [34]. Memory Th2 cells convert to iTreg when treated in vitro with TGF-β and in response to blockade of IFN-γ and IL-4 signalling [35]. Investigators have developed mathematical models that predict how the additive integration of signals to T cells from cytokines determine the fate outcome of CD4+ve T cells [36]. ILCs are first line against pathogens and environmental influences drive a change from one phenotype to another. For example, Group 2 ILCs residing in the lungs of mice become group 1 ILCs upon infection by influenza virus and *Staphylococcus aureus* and exposure to cigarette smoke [37].

From all of the above, cells are much more versatile/plastic than we previously imagined. Developing HSPCs can change their first-choice pathway to a different pathway and the phenotype of at least some of the mature immune cells is flexible throughout their lifespan. Perhaps the extent that investigators rigorously defined cell surface phenotypes and other functional attributes misled us to view acquired characteristics as somewhat fixed. By contrast and as early as 1987, Shankland commented that it would be very surprising if cells progressing along a lineage pathway were not able to follow a different pathway in response to a particular circumstance. Shankland observed that neurons and epidermal specialisations normally descend in the embryo of the leech from the p blast cell and that ablation of the P cell line led to neurons and epidermal specialisations arising from a different precursor—the o blast cell [38]. In this case, the o blast cell adopts an alternative cell lineage. It would be challenging to investigators to chronicle the extent that developing murine HSCPs can step sideways in vivo because of the need to ablate, for example, just one population of lineage affiliated HSPCs. The identification of these cells has blurred the demarcation between HSCs and HPCs and we might argue that the range of cell types seen in the various colonies arising from single HPCs dispersed in semi-solid medium reflects an intrinsic capacity to shuffle sideways. In keeping with the continuum model, the founder cells divert to lineages that are adjacent to one another rather than in a random manner.

Haematopoietic stem cells, progenitor cells and end cells moving from one cell lineage/sub-type to another appears to involve “switches” that integrate genetic and environmental influences on a cell’s phenotype. As to the role of environment influences, the versatility may be adaptive to meet the demands of an organism for a particular type of cell. Additionally, central to evolutionary biology is explaining how novel traits/cell types arise [39]. The inherent versatility of cells and the extent that environmental conditions can guide a cell towards an alternative developmental pathway might underlie the evolution of new patterns of traits/cell types.

## 4. The Bulk of Leukaemia Cells belong to just One Cell Lineage

A feature of the bulk of leukaemia and cancer cells is that they resemble just one normal cell type and we categorise the various leukaemias, and other cancers, by this means. However, most, if not all, leukaemia and other cancers arise from a tissue specific stem cell [40]. Chronic myeloid leukaemia (CML) and acute erythroid leukaemia both arise from a haematopoietic stem cell, but strikingly the leukaemia cells belong to the neutrophil and erythroid pathways respectively (reviewed in [41]). We know quite a lot about many cancers, in particular the oncogenic insults to normal cells leading to disease. A question that has received much less attention is how cells are “dumped” down one developmental pathway by HSCs in the case of CML and acute erythroid leukaemia. To put this question another way: Regarding the many leukaemias and cancers that arise in a tissue-specific stem cell, why is the hierarchy of the partially or fully differentiated malignant cells generated not a mixture of cell types?

Investigators and clinicians view childhood B-cell precursor acute lymphoblastic leukaemia (B-ALL) as arising in a committed B-lineage cell [42]. However, HSC-like cells expressing the stem cell marker CD34 and lacking B-lineage markers recreate the disease in mice indicating a primitive cell origin for some types of B-ALL [43]. Infant B-ALL (iB-ALL), at <1 year of age, is a different disease with a more aggressive presentation and remains fatal as compared to a current 5-year survival approaching 90% for childhood B-ALL. Comparison of the gene expression signature of iB-ALL blasts with those of human foetal live HSPC populations showed that that the iB-ALL blasts resemble the most immature foetal liver HSPCs, similar to that of Lin^−ve^ CD34^+ve^ CD38^−ve^ CD19^−ve^ foetal HSPCs as opposed to the distinct signature of foetal liver B-cell committed progenitors. These findings suggest that iB-ALL arises in a primitive foetal liver HSPC that is developmentally prior to the “pre-VDJ” stage of B-cell development [44].

For other leukaemias, findings support an origin in HSCs rather than the long-held viewpoints of a lineage-affiliated cell. The genetic alterations for T cell lymphoma [45], follicular lymphoma [46,47] and hairy cell leukaemia [48] trace back to the HSC stage of development. Investigators categorise acute promyelocytic leukaemia as a myeloid progenitor malignancy [49]. By contrast, the fusion PML-RARα oncoprotein, arising from the t(15,17) translocation, is characteristic of acute promyelocytic leukaemia and both markers are present in CD34+ve CD38-ve HSC-like cells from patients [50]. A customary view is that chronic lymphocytic leukaemia (CLL) is a disease of B-lymphocytes, but patients’ HSCs that express lymphoid genes indicate that a self-renewing HSC is the primary target cell in the pathogenesis of CLL. Additionally, HSCs from patients with CLL produce a high number of polyclonal B-cell progenitors [51,52]

## 5. Oncogenic Programming of Stem and Progenitor Cells to a Single Cell Lineage

The *LMO2* gene encodes the LIM-only transcription factor (Lmo2) that also has a role in promoting DNA synthesis and progression from the G1 phase to S phase of the cell cycle in haematopoietic cells [53]. There is often activation of transcription factors in leukaemias and *LMO2* is a target of activation via chromosomal translocations. Strikingly the chromosomal translocations that activate the *LMO2* gene occur only in T-cell leukaemias. In other words, the *LMO2* gene when abnormally expressed exclusively drives T-cell malignancies [54].

Despite the T-cell exclusivity of the oncogenic nature of *LMO2*, McCormack and colleagues have argued from conditional knockout of expression of mouse *LMO2* that Lmo2 does not have a mandatory role in normal T cell development [55]. They expressed the Cre recombinase under the control of the promoters of the lymphoid-specific genes Rag1, CD19, and Lck to delete the expression of LMO2 in specific cell types. The deletion of *Lmo2* was efficient within bone marrow progenitors of lymphocytes and there was no disturbance to either T- or B-cell development. However, Lmo2 is necessary for early stages of haematopoiesis because homozygous mutant *LMO2*^−/−^ mouse embryonic stem cells do not contribute to haematopoiesis in adult chimeric mice [56]. Lmo2 plays a role in red cell development in the embryo yolk sac (primitive erythropoiesis) and might have a role in adult haematopoiesis at a stage of development that is earlier than lymphoid progenitors, and in HSPCs that is of interest as follows.

For human leukaemias, it is difficult to ascertain whether the transforming action of Lmo2 is within HSCs or at some later lineage-committed stage of development. This is because there is preservation of the action of Lmo2 that leads to programming of the malignant cell fate in the stem cells that maintain the leukaemia, their immediate progeny and the partially differentiated leukaemia cells that are the bulk of a patient’s leukaemia cells. The precise role of Lmo2 at each of these different developmental stages of the leukaemic cell hierarchy is therefore difficult to unravel by analysing patients’ cells.

**Figure 3:**
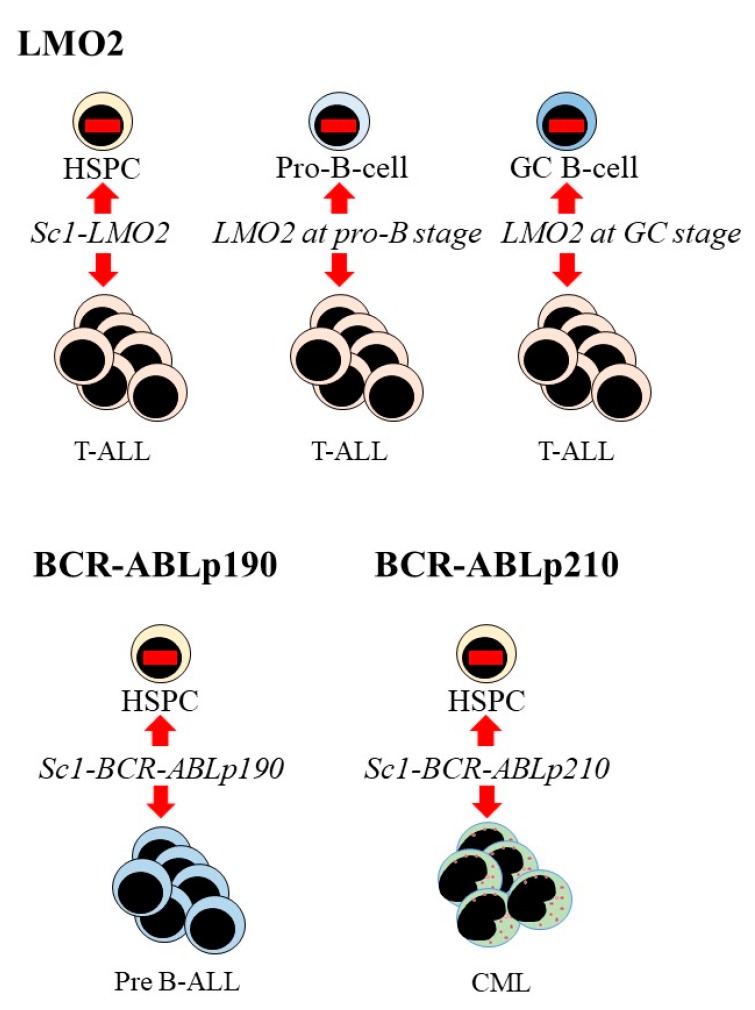
Expression of LMO2, BCR-ABLp190 and BCR-ABLp210 leads to T-cell, B-cell and myeloid leukaemia respectively. Targeted expression of *LMO2* to haematopoietic stem and progenitor cells (HSPC) was via Sc1-LMO2. Expression at the pro-B cell stage of development was via crossing *Rosa26-Lmo2* and Mb1 mice to delete the stop cassette at the pro-B-cell level (see main text). Crossing conditional *Rosa26-Lmo2* mice with the *Aid-Cre* strain induced Lmo2 expression in GC cells (see main text). These enforced expressions led to T-acute lymphoblastic leukaemia (T-ALL). Targeted expression of *BCR-ABLp190* and *BCR-ABLp210* to HSPC, via Sc1-LMO2, led to pre-B acute lymphoblastic leukaemia and chronic myeloid leukaemia respectively.

To circumvent the caveat to studies of patients’ cells, investigators enforced expression of Lmo2 in mouse HSPCs and B cells at various stages of their development and only T cell leukaemias arise (Figure 3). Targeted and restricted Lmo2 expression in mouse HSCs, by placing *LMO2–TdTomato* cDNA under the control of the stem-cell-specific Sca1 promoter, resulted in a highly disseminated human-like T acute lymphoblastic leukaemia (T-ALL), with the resulting leukaemia cells lacking expression of Lmo2. To achieve expression of Lmo2 at the pro-B cell stage of development, the investigators crossed *Rosa26-LMO2* and Mb1 mice to delete the stop cassette at the pro-B-cell level, via the Cre recombinase driven by the promoter from the Mb1 locus encoding the immunoglobulin-associated alpha chain Cd79a. Activation of *LMO2* at pro-B cell stage of B-cell development had a minimal effect on B-cell development and instead reprogrammed B-cells leading to the development of aggressive T-ALL. Crossing conditional *Rosa26-LMO2* mice with the *Aid-Cre* strain, which expresses Cre recombinase in germinal center (GC) B cells, led to Lmo2 expression in GC cells. Expression of Lmo2 in GC B cells (see Figure 2 legend) did not result in B-cell malignancies and again the mice developed aggressive T-ALL. In all the above instances, Lmo2 expression has defined/fixed the leukaemia T-cell identity of different target cells and their progeny through reprogramming [57].

Two forms of the *BCR-ABL* oncogene encode the fusion proteins BCR-ABLp190 and BCR-ABLp210 that are associated with different forms of leukaemia. The BCR-ABLp190 occurs commonly in Ph+ve ALL and occasionally in acute myeloid leukaemia. The BCR-ABLp210 occurs in patients with CML, and in acute lymphoid and myeloid leukaemias [58]. There are healthy individuals that have the CML-associated *BCR-ABLp210* transcripts, with reported incidences ranging from 10% to 30% of adult peripheral blood samples tested (reviewed in [59]). Similarly, investigators have found cells that have the BCR-ABLp190 lesion in neonatal cord blood and the majority of carriers did not develop B-cell ALL [60,61]. The presence of single *BCR-ABLp190* mutation seems to be insufficient to cause B-cell ALL, other cooperating mutations are required and the cells that just have the mutation are premalignant.

Gene targeting experiments have addressed the roles of BCR-ABLp190 and BCR-ABLp210 in the genesis of leukaemia (Figure 3). Investigators modelled the role of BCR-ABLp190 in mice and expression was restricted to HSPC by means of Sca1-BCR-ABLp190. The mice developed B-ALL at a low penetrance. The leukaemic blast cells were transcriptionally pro-B/pre-B cells, resembled the human disease and did not express BCR-ABLp190. The oncogenic action of BCR-ABLp190 is therefore hit-and-run. A short disease latency and 90% incidence was observed for double Sca1-BCR-ABLp190 + *Pax5*^+/−^ mice and an accumulation of genomic alterations was observed in in the remaining wild-type *Pax5* allele. A number of metabolic genes (*IDH1, G6PC3, GAPDH, PGK1, MYC, ENO1, ACO1*) were upregulated in the Pax5-deficient leukemic cells, and human leukeamia cells have a similar metabolic signature. This finding led investigators to propose that disease occurs upon reduced Pax5 functionality and that metabolic reprogramming combined with Sca-restricted BCR-ABLp190 expression leads to successful transformation [62]. The Pax5 B-cell specific transcription factor is present in pro-B cells and in all further stages of B cell development until the plasma cell stage. As to disease requiring reduced Pax5, it therefore seems unlikely that disease occurred in a committed Pax5+ve pro-B/pre-B cell that had acquired stem cell-like properties to sustain disease. Additionally, and in keeping with the stem cell-origin theory of cancer [40], there is no demonstration of an oncogene reverting a committed cell to a stem cell-like state and committed epithelial cells do not give rise to the malignant squamous cell carcinomas [63]. An alternative view regarding disease initiation is that Sca1-restriction of BCR-ABLp190 expression to HSPCs has “wired” HSPCs that do not express Pax5 to a leukaemic phenotype including imposing B-cell lineage development.

In contrast to BCR-ABLp190, transient expression of BCR-ABLp210 restricted to the HSPC compartment of mice led to mature myeloid leukaemia [64,65]. Again, the disease in mice resembles human CML. CML stem cells are not dependent on the BCR-ABLp210 tyrosine kinase activity because the clinical use of ABL kinase inhibitors does not cure the disease. The disease returns upon cessation of treatment due to the persistence of CML stem cells or the development of resistance [66]. As to the persistence of disease following kinase inhibition, an oncogenic activity of BCR-ABLp210 is therefore distinct from the ABL kinase activity and a postulate regarding this additional activity is that BCR-ABLp210 instruct myeloid development of the malignant HSC [65].

## 6. How Might Hard Wiring of Leukaemia Stem Cells to One Cell Lineage Occur?

For HSCs, the oncogene-mediated hard wiring to a cell lineage might occur within HSCs or a later stage of affiliation of HSCs to a cell lineage. As outlined above, HSCs are sufficiently versatility for the programming to occur. In the case of hard wiring of an offspring of HSCs, the oncogene action might synergise with a lineage-affiliate event(s), which is either intrinsic to the cell or driven externally. Fixation of a lineage affiliation might occur via wiring of the network of transcriptional factors. Alternatively, certain oncogenes might either reshape the epigenome or remodel the architecture of chromatin. We favour the view that oncogenic insults to the genome shape the epigenome [57].

The transcription factor Lmo2 is at the very core of the identity of HSPCs. Lmo2 is required for TAL1 to bind to DNA and this is important at the haemangioblast stage of haematopoiesis to position the TAL1/Lmo2/LDB1 complex to regulatory elements to establish the developmental program and initiate definitive haematopoiesis [67]. In keeping is that in chimeric mice Lmo2^−/−^ cells do not contribute to definitive haematopoiesis [56]. Even so, it is important to separate the function of Lmo2 in the haemangioblast to establish haematopoiesis and a role in the T-ALL arising from the enforced oncogene expression. A possibility is that oncogene enforced Lmo2 expression within HSCs leads to over-expression that persists when these cells become T-cell lineage affiliated. In this case, the abnormal expression of Lmo2 together with transcription factors that play an obligatory role during T-cell development, for example E2A-Heb heterodimers [68], may be important, and Lmo2 endows stem cell attributes as to driving self-renewal of thymocytes [69].

**Figure 4:**
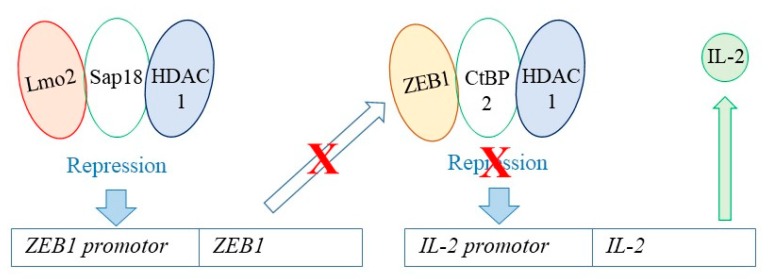
Links between Lmo2, epigenetic regulation and Il-2 gene expression. Lmo2 recruits Sap18 and histone deacetylase 1 (HDAC1) to repress transcription of ZEB1 via histone deacetylation at the ZEB1 promotor. ZEB1 cooperates with CtBP2 and HDAC1 to repress *IL-2* gene expression. Whereupon oncogenic Lmo2 represses the transcription of ZEB1 there might be expression of IL-2.

Lmo2 plays a role in chromatin remodelling. As considered above, some haematopoietic cytokines instruct cell lineage and ensure the survival and proliferation of lineage-affiliated cells. There is an intriguing link between Lmo2 expression and the epigenetic regulation of the expression of the T-cell associated cytokine IL-2 (Figure 4). Oncogenic Lmo2 recruits Sap18 and histone deacetylase 1 (HDAC1) and this epigenetic regulatory complex represses transcription of ZEB1, a member of the zinc finger-homeodomain family of transcription factors, by means of histone deacetylation at the ZEB1 promotor and chromatin remodelling. Epigenetic dysregulation of ZEB1 is involved in the genesis of T-ALL and ZEB1 plays a role in normal T-cells by cooperating with CtBP2 and HDAC1 to repress the expression of the *IL-2* gene [70]. Whereupon oncogenic Lmo2 represses the transcription of ZEB1, there might be expression of IL-2 to drive either T-cell expansion or effector T-cell differentiation [71]. Suppression of ZEB1 expression also affects signalling by transforming growth factor-β1 because ZEB1 has an enhancing role as revealed by ectopic expression. Accordingly, ZEB1 is a candidate tumour-suppressor gene and down-regulation contributes to resistance to TGF-β1-mediated growth suppression in adult T-ALL and lymphoma [72]. Lmo2 and ZEB1 can also influence the stem-ness of cells. Overexpression of Lmo2 leading to suppression of ZEB1 expression within the leukaemic T cell lines Jurkat and MOLT4 increased the appearance of cells expressing stem cell properties (CD117+ve and CD34+ve) and rescue of ZEB1 expression attenuated this effect [73].

From all of the above it is important to bear in mind that we should not view Lmo2-provoked changes to the transcription factor network and the epigenome as separable. Additionally, consideration of Lmo2-provoked changes to the levels of and signalling by haematopoietic cytokines are important because such is crucial to the normal conduct of haematopoiesis. There are intriguing links between Lmo2 expression and changes to the transcription factor network, the epigenetic status of cells and the levels/actions of cytokines. Presently, we do not know how events might conspire to “fix” HSCs and their progeny to the T-cell lineage, if this is the case, and lead to overt leukaemia.

When pieces of chromosomes 9 and 22 change places to give rise to the shorter Ph 22 chromosome, the ABL gene (on chromosome 9) joins to the BCR gene (on chromosome 22) to form the BCR-ABL fusion gene. The encoded hybrid protein is a constitutively active tyrosine kinase and this is important as clinicians use tyrosine kinase inhibitors to treat CML successfully. So far, it is hard to see how the two forms of BCR-ABL forms clearly associate with different leukaemias and there are two possibilities. One is that the intrinsic leukaemogenic activity within the cell that has the translocation, and is expressing BCR-ABL, is different for the two forms. Alternatively, the activity of the two forms is the same and the expression of each form is somehow restricted to different haematopoietic cell lineages because a given lineage favours a particular breakpoint during the formation of the Ph chromosome.

A hypothesis is that BCR-ABLp210 “wires” the transformed HSC and its progeny cells to the myeloid differentiation pathway by fuelling epigenetic changes. Reduced representation bisulfite sequencing to interrogate the DNA methylation landscape in HSPCs (Sca1+ve Lin−ve) from wild type and Sca1-BCR-ABLp210 transgenic mice revealed global hypomethylation within the HSPCs from the Sca1-BCR-ABLp210 mice, and in the mature myeloid cells despite their lack of expression of BCR-ABLp210 [65]. The broad loss of methylation was at CpG islands. Investigators have shown that there is over expression of DNA methyltransferas (DNMT) genes, including DNMT1, in myeloid leukemias [74]. Within HSPCs from the Sca1-BCR-ABLp210 mice, as compared to wild type mice, *Dntm1* gene expression was upregulated, *Dmt3b* downregulated and there was no change in *Dnmt3a* [65]. The finding that Sca1-Dnmt1 mice develop myeloid malignancies with marked expansion of granulocytes in the blood and bone marrow supports *Dntm1* expression playing an important role in CML. All of the above provides support to the notion that *BCR-ABLp210*-provoked changes to the epigenome “fix” the lineage identity of the transformed HSC and its progeny leukaemia cells to that of myeloid development [reviewed in 65].

## 7. Concluding Remarks

The arguments presented above centre around the extent normal and leukaemia stem cells and their progeny can cross the “boundaries” that delineate one cell lineage from another as cells develop along a pathway. We have argued that this is different for HSCs and leukaemia stem cells (LSCs) and their progeny whereby LSCs and their progeny do not have the capacity to follow an alternative pathway after they have affiliated to a cell lineage. There is good evidence to support the view that normal HSCs and their progeny are indeed versatile and experimental evidence to support oncogene priming of the epigenome of leukaemia cells such that they adopt one cell lineage. The pathological cell identity established is specific for each different oncogene. We believe that hard wiring of transformed cells to a cell lineage is a characteristic of many cancers because stem cells are largely the origin of “successful” cancers. The instructive cytokines provide a unique opportunity to test whether leukaemia cells are hard-wired which is important to the way we treat cancer. We can erase epigenetic insults to the genome and indeed the HDAC inhibitors vorinostat, romidepsin and belinostat have approval for treating some T-cell lymphoma patients and panobinostat for treating multiple myeloma patients. Other inhibitors are in clinical trial for the treatment of leukaemias and solid cancers. These agents have what seems a bright future as one of the tools to fight against cancer.
